# Transforming an Academic Military Treatment Facility Into a Trauma Center: Lessons Learned From Operation Iraqi Freedom

**Published:** 2009-07-24

**Authors:** Eric A. Elster, Jonathan P. Pearl, John W. DeNobile, Philip W. Perdue, Alexander Stojadinovic, William A. Liston, James R. Dunne

**Affiliations:** ^a^ Department of Surgery, National Naval Medical Center, Bethesda, MD; ^b^ Department of Surgery, Uniformed Services University of the Health Services, Bethesda, MD; ^c^ Combat Casualty Care, Naval Medical Research Center, United States Navy, Silver Spring, MD

## Abstract

**Background:** To manage the influx of patients with predominately extremity injuries from Operation Iraqi Freedom (OIF), our center was required to transform from a nontrauma academic hospital to a trauma hospital by using a multidisciplinary approach. **Study Design:** A retrospective chart review was performed of casualties from OIF who were received over 14 months. **Results:** A total of 313 casualties were received. The average number of admissions was 16 per month, except during November 2004, when there were 88 admissions over 7 days. The mean ISS for all patients was 14.1 ± 10.3. A total of 113 patients (36%) required admission to the intensive care unit for an average of 7.5 ± 5.2 days. The mean interval between injury and arrival in the continental United States was 6.5 ± 4.6 days. Most casualties suffered multisystem trauma, with extremity injuries predominating. The multidisciplinary approach to casualty care consisted of several meetings a week and included everyone involved in caring for these combat casualties. **Conclusions:** A multidisciplinary approach transformed an existing medical center into a trauma receiving hospital capable of managing and maintaining a surge in patient admissions resulting in minimal morbidity and mortality. This model further supports a multidisciplinary approach to trauma care and could serve as a guideline for transforming existing medical centers into trauma receiving hospitals to deal with patient overflow in the event of future civilian mass casualties.

The nature and number of battlefield casualties from all phases of Operation Iraqi Freedom (OIF) have placed a tremendous strain on both deployed and stateside Department of Defense medical assets. As part of military doctrine, hospitals that previously have served as tertiary care centers managing the occasional injury from peacetime operations but without maintaining trauma services have transformed into facilities treating numerous patients with complex injuries. The vast majority of US Marines and Navy personnel injured have been treated at the National Naval Medical Center (NNMC).^1^ Faced with the considerable influx of OIF casualties, the NNMC transformed from a tertiary referral hospital without sustained trauma care into a trauma receiving to meet this wartime need. Our experience may provide a model for instituting trauma care in a community hospital in the event of civilian mass casualties that could overwhelm the trauma system in place.

Casualties from OIF resulted in an increase in hospital admissions and operative caseload that required complex management. As with most nontrauma centers, prior to the conflict such cases were handled by admissions to separate services without integration of care. Early on in our experience, we determined that this model would not work for the sustained influx of casualties, especially with respect to patient disposition and coordination of multiple procedures. Therefore, we determined the increase in workload and the resulting coordination of care that was required demanded the institution of an integrated multidisciplinary approach to these combat casualties as seen in the American College of Surgeons (ACS)–accredited trauma centers.

The NNMC is considered a level V facility in the current military medical system. These 5 echelons of care encompass treatment from the battlefield to final disposition in the United States.^2^ In this system, first responder capability (level I) is composed of self- or buddy-care and corpsman aid, in addition to initial management using ATLS-based principles. Forward resuscitative care (level II) provides initial damage control and stabilization. This care is provided by the forward resuscitative surgical support teams attached to Marine units. En route care moves patients from these forward treatment areas to a theater hospitalization capability (level III), which can provide subspecialty surgical services as well as inpatient care. En route care also encompasses movement to fixed facilities outside the theater of operations (level IV), such as Landstuhl Regional Medical Center (LRMC) in Germany, through which most OIF casualties are routed. Final patient care and disposition for those requiring long-term treatment are provided at definitive care medical centers such as NNMC (level V) in the United States. Operative interventions take place at levels II, III, and IV, and often patients will have undergone 1 or 2 operations prior to arrival at level V. The efficiency of the medical evacuation system has allowed for some of these casualties to reach definitive care medical facilities in the United States as soon as 48 hours, thereby requiring acute management of these patients.

Prior to OIF, the NNMC had served as a 250-bed tertiary university (Uniformed Services University)–affiliated teaching hospital that offered general and subspecialty surgical and nonsurgical care to DOD healthcare beneficiaries. While this patient population provided a diverse caseload prior to OIF, the NNMC did not function as the ACS Committee On Trauma verified trauma center. As such, no system-based integrated trauma care was provided and beneficiaries sustaining traumatic injuries in the NCA were cared for at local ACS-approved trauma centers. Our objective in this report is to highlight our experience in dealing with casualty care and demonstrate how existing models of multidisciplinary care can be rapidly adopted by civilian centers if circumstances overwhelmed the standard trauma systems.

## METHODS

A retrospective database of clinical and logistical information was collected on all OIF casualties received at the NNMC between March 24, 2004, and May 31, 2005. Permission to conduct this study was granted by the local institutional review board.

Database elements included demographics, transport interval between injury and arrival at the NNMC, intensive care unit (ICU) admissions, injury severity, operations performed, length of stay, complications, and disposition. Injury Severity Scores (ISS) were calculated using standard means upon admission. ISS is a standard metric used to quantify injury severity in trauma patients and correlates with mortality, morbidity, and hospitalization time after trauma.

The dedicated trauma team was composed of existing personnel in the Department of General Surgery: an attending surgeon, a chief resident and junior resident, and at least 1 intern. The team conducted morning rounds, spent the bulk of the day in the operating room (OR), and then assembled at a daily afternoon multidisciplinary meeting. While morning rounds focusing on the surgical services, the afternoon meeting coordinated care as outlined below.

Disjointed care was minimized by convening these daily multidisciplinary team meetings supervised by the attending surgeon. Also in attendance were representatives from each of the surgical and medical teams caring for the patients. In addition, members of the ancillary staff reported on the progress of each patient. Each wounded service member was assigned a nurse case manager and social worker who were instrumental in planning disposition and follow-up care. A representative from the OR and the surgical ward also attended to allocate and maximize resources. In addition to discussing the medical needs of the patient, issues surrounding family members were also discussed.

The purpose of the meeting was to enhance communication, coordination and continuity of care, allocation of resources, and disposition planning. Each patient was discussed and daily plans were made according to their specific needs, taking into account the needs of their family as well. Often information regarding discharged patients was discussed, thus providing a forum for quality assurance and planning of future medical care.

## RESULTS

### Demographics and patient flow

A total of 313 casualties from OIF were admitted between March 24, 2004, and May 31, 2005. All but 2 were male, with a mean age of 24.1 ± 4.2 years and a mean ISS of 14.1 ± 10.3. A total of 113 patients (36%) required admission to the ICU for an average of 7.5 ± 5.2 days. The average length of stay at the NNMC was 14.1 ± 7.1 days. The mean interval between injury and arrival in the continental United States was 6.5 ± 4.6 days (Table 1).

**Figure 1:**
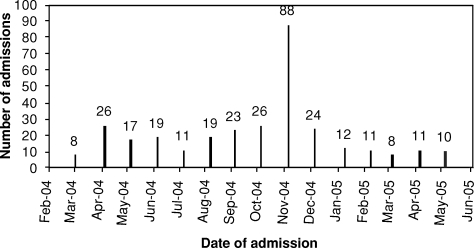
Monthly admissions.

Medical evacuation flights typically arrived from Europe 2 to 3 times per week. Excluding November 2004, the NNMC received an average of 16.1 ±6.0 admissions per month (Fig 1).

During November 2004, the Marine Corps launched an offensive on the city of Fallujah, located west of Baghdad. During that month, and largely as a result of the siege on Fallujah, the NNMC experienced a substantial influx in casualty admissions to a total of 88 over a 7-day period, roughly 4 times the monthly average of 16 admissions. The average ISS for these patients was 12.8 ± 10.4, with 28 having ISS > 16 (32%). This surge in patients included 27 ICU admissions with an average ICU stay of 4.3 ± 2.1 days. Average length of stay for this entire cohort of casualties from Fallujah was 18 ± 4.6 days. The overall number of operations performed on these 88 patients was 226, of which 184 were performed in the first week (Table 2). This required almost continuous OR utilization and was carried out with only a temporary reduction in scheduled cases, all of which were rescheduled and performed the next week.

Throughout most of the study time period, the NNMC ran 12 ORs daily, with at least 1 devoted to care of the casualties. Elective operations have continued, with priority given to casualties in need of urgent care. The volume and acuity of patients received during November 2004 necessitated that 4 ORs be devoted to casualty care for at least 12 hours per day. Despite this, less than 5% of elective cases were postponed.

### Injury patterns and operative care

During OIF, the insurgents' predominant use of improvised explosive devices as weaponry as well as the use of improved body armor has resulted in distinctive injury patterns.^3^,^4^ Polytrauma has been common, with 576 body regions injured in the 313 patients. Upper and lower extremity injuries accounted for 350 of the 576 injuries (61%) followed by head and neck soft tissue injuries (16%) and brain injuries (10%). There were 47 abdominal injuries (8.1%) and 28 chest injuries (4.9%).

**Figure 2:**
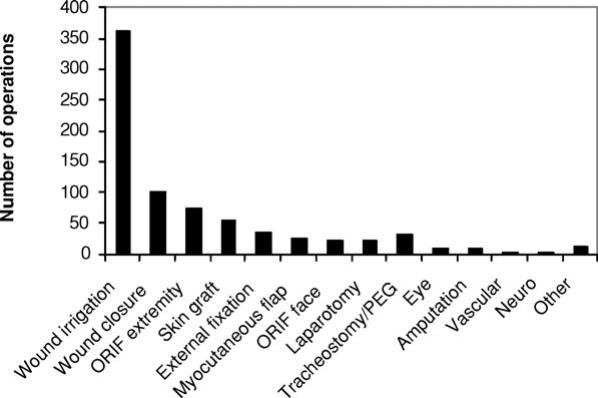
Operations performed. ORIF indicates open reduction and internal fixation; PEG, percutaneous endoscopic gestrostomy.

Nearly 75% of patients included in this cohort required an operation during their hospital stay. A total of 786 operations were performed on 230 patients. Wound irrigation, debridement, and placement of vacuum-assisted wound closure devices amounted to 362 (46%) of the operations. Other wound management including delayed primary closure, split thickness skin grafting, and myocutaneous flaps yielded 103, 56, and 26 operations, respectively (Fig 2).

### Multidisciplinary care

Weekly video teleconferences with providers in theater and at the LRMC allowed for both feedback and planning. This was critically important as it allowed communication between facilities prior to the institution of sophisticated record keeping, therefore providing information regarding previous treatments and mechanism of injury. In addition, this dialogue allowed for feedback on patient care to the LRMC and permitted that facility to change care practices when needed.

Caring for the casualties required multidisciplinary cooperation with participation of multiple services. An aggressive screening program was instituted by behavioral health to identify even the most mild of traumatic brain injuries that has been characterized by some as the signet injury from this war. This screening program was applied to all patients returning from OIF/Operation Enduring Freedom (OEF) and included various neuropsychological tests.^5^ The results of these tests were discussed with neuropsychology, behavioral health, and the entire multidisciplinary team. On the basis of these recommendations, the patient was referred for regular outpatient follow-up, intensive outpatient treatment, or intensive inpatient rehabilitation. Extremity wound care, which consumed the bulk of the operative efforts, was shared by orthopedic surgeons, general surgeons, and plastic surgeons. All patients were evaluated by a team of pain specialists to optimize their analgesia. Skin grafting and local tissue transfers were performed by both orthopedic surgeons and general surgeons, while complicated tissue transfers and free flaps were reserved for the plastic surgeons.

### Morbidity and mortality

Postinjury complications occurred in 20% (*n* = 64) patients while admitted to the NNMC. The most common morbidities were wound infection (*n* = 15), retained hemothorax (*n* = 6), and thrombosis of vascular grafts (*n* = 5). Thromboembolic complications were rarely detected (deep venous thrombosis [*n* = 3], pulmonary emboli [*n* = 2]), although diagnostic examinations to detect clinically occult thromboembolic complications were not routinely instituted.

The in-hospital mortality rate was 1.2% (*n* = 4). The earliest mortality occurred 2 days after admission, and the latest occurred on hospital day 9. The causes of death were exsanguinations (*n* = 2), sepsis (*n* = 1), and massive head injury (*n* = 1).

### Disposition

The average length of stay at the NNMC was 14.1 ± 7.1 days. Two thirds (204/313) of the casualties were granted convalescent leave at their homes of record, and 43 patients returned to their parent commands. A total of 38 patients required inpatient traumatic brain injury or physical rehabilitation at one of the newly designated Veterans Affairs polytrauma centers. Another 28 patients required amputee care at Walter Reed Army Medical Center (Table 3). This disposition of patients was not final; ongoing communication between these centers and the NNMC by using a weekly teleconference allowed for patient transfer between specialty centers and return to the NNMC for follow-up care and surgery when needed.

## DISCUSSION

During OIF, the increased tempo of operations, the sheer number of injuries, the complexity of these injuries, and the expedited transit to stateside care have placed unique demands upon military treatment facilities.^6^ Treatment of these patients has required new paradigms throughout all levels of care.^6^–^9^ To meet the challenges associated with the care of these war fighters, we have adopted a multidisciplinary approach. Our experience may provide a reasonable model for community hospitals in caring for mass casualties during a civilian disaster.

Over the 15-month time period of this study encompassing a portion of OIF, we have admitted 311 patients, of which approximately one third have required ICU admission. In addition, during times of intense ground combat in theater, such as seen in November 2004 during the siege of Fallujah, we saw a dramatic surge in critically wounded patients.

Our multidisciplinary approach grew out of the initial influx of patients when we determined that the current system of admission to various services resulted in miscommunication and delayed disposition and therefore was not ideal for the care of these complex patients. With a general or trauma-trained surgeon functioning as a team leader, a team composed of orthopedic surgeons and other key surgical and nonsurgical subspecialists was supported by integrated OR scheduling, nursing, pain management, social work, and physical and occupational therapy. This core group met daily to ensure that the continuum of care stretched from admission to disposition: either discharge or transfer to other facilities. This also included arranging follow-up care at the NNMC and transfer between specialty centers (i.e., patients who required amputee care as well as neurological rehabilitation at different centers). This multidisciplinary care was extended beyond the NNMC to reach out to affiliated institutions at all levels in the treatment chain by the use of the video teleconference, allowing feedback and integration of care.

Using this integrated approach, a total of 786 operations were performed on the majority of these patients. As has been reported previously, extremity injuries predominated, but multisystem injury was common with a total of 576 injured body regions in the 313 patients.^1^,^9^–^11^ These injuries were often complex and required the coordinated operative care and perioperative treatment that the integrated approach provided. The majority of these operations involved wound irrigation and debridements, supplemented by delayed primary closure, skin grafting, and myocutaneous flap closure. These operations were performed by general and orthopedic surgeons with plastic surgery support for complicated tissue transfer coverage as well as free flap closure. The operative load required for these cases, which often included multiple operations per patient, required dedicated ORs that functioned continuously during high-volume periods. Throughout this entire period, the NNMC continued with a full elective and emergent operative load with less than 5% of elective cases rescheduled.

The most taxing phase of care during this time period came with the surge of casualties (*n* = 88) from fighting in and around Fallujah. The capability of this multidisciplinary approach to deal with a surge of severely injured group of patients (32% with ISS > 16) requiring intensive care (32% admitted to ICU for an average of 4.3 ± 2.1 days) and operative procedures (184 operations in the first week, 226 overall) compares with the Israeli experience in dealing with terror-related injuries in an integrated trauma system with short transit times.^12^–^13^ The only US correlate to such an influx of severely injured patients is the experience after the Oklahoma City bombing in 1995 and the destruction of the World Trade Center on September 11, 2001.^14^–^16^ In both of these instances, a similar number of injured patients were admitted to the hospital (112 at St. Vincent's in New York and 72 at Baptist Medical Center in Oklahoma City) with many more screened in the emergency department. Consistent with the nature of the attack in each of these instances and the initial screening and treatment of OIF casualties, the acuity of patients admitted was lower than the patient load seen at the NNMC with only 5% to 8% admitted to the ICU and only 5% of patients with ISS > 15.^14^–^16^ In addition, after the initial surge these patients from domestic terror-related incidents required less operative care (13 operations at St. Vincent's and 25 patients at Baptist Medical Center). However, the transit time from theater to the NNMC did allow for some element of planning. Therefore, it appears that although the acuity of care from previous incidents was less than seen at the NNMC, an integrated approach as seen at St. Vincent's, Baptist Medical Center, and the NNMC can effectively manage such large numbers. Furthermore, in the advent of such a mass casualty scenario where primary trauma centers are overwhelmed, secondary facilities with limited trauma services may be required to adopt such practices.

Despite the destructive nature of these wounds and the initial contamination from the field environment, our morbidity rate was acceptable. Of the 64 perioperative complications seen in this patient population, the most common were wound infections. Other complications including thromboembolic and retained hemothorax were rare. The overall mortality was 1.2%, with 2 patients dying from exsanguination secondary to ruptured pseudoaneurysms despite heroic efforts. Although no comparative data have been reported, this overall low mortality was expected given the delay in casualty transit from theater. After an average hospital stay of 1 week, the majority of patients (80%) were discharged. The remaining 20% of patients required further care at other institutions including Veterans Affairs Hospitals. The transition to these facilities was enhanced by the multidisciplinary approach and collaboration with the Department of Veterans Affairs for patients requiring rehabilitation, especially those with head injuries.

These data suggest that an integrated multidisciplinary facilitates efficient distribution of resources, especially in institutions in which disaster plans focus on the initial surge of patients and not on sustainability.^1^,^17^ This was achieved by improved communication between various specialties that reduced multiple trips to the OR by coordinating specialty care and fostered a continuum of care that extended beyond discharge. The expedited transfer of casualties from the battlefield as well as the need for sustained care may provide a model for the civilian experience with terror-related incidents and other mass casualty events. This is in sharp contrast to previous military conflicts in which casualties arrived in the United States primarily for convalescent care. The planning for such contingences should start early and the use of existing multidisciplinary approaches such as highlighted herein should be emphasized. The ability to efficiently transform into an institution that can provide sustained mass casualty care without impairing primary services is crucial for both military and civilian institutions.

## ACKNOWLEDGMENTS

This work was supported and funded by work unit number: 604771N.0933.001.A0604.

The multidisciplinary care of these patients would not have been possible without the dedicated efforts of everyone at the NNMC. Both civilian and military personnel have rendered skilled and compassionate care for these casualties. All of our efforts are dedicated to those who have been placed in harm's way for the good of our nation.

## Figures and Tables

**Table 1:** Patient demographics*

Variable		*n*
Mean age, y	24.1 ± 4.2	313
Mean Injury Severity Score	14.1 ± 10.3	313
ICU admission	36%	113
Mean ICU LOS, d	7.5 ± 5.2	113
Mean hospital LOS, d	14.1 ± 7.1	313

*ICU indicates intensive care unit; LOS, length of stay.

**Table 2:** Patient demographics for Fallujah Siege*

Variable		*n*
Mean Injury Severity Score	12.8 ± 10.4	88
ICU admission	27%	24
Mean ICU LOS, d	4.3 ± 2.1	24
Mean hospital LOS, d	18.0 ± 4.6	88
Operative procedures		226
Mortality	3.4%	3

*ICU indicates intensive care unit; LOS, length of stay.

**Table 3:** Patient disposition

Location	*n*	%
Home (convalescent leave)	204	65.2
Parent command	43	13.7
Veterans Affairs Hospital	38	12.1
Military Hospital	28	8.9
